# Patterns of geohelminth infection, impact of albendazole treatment and re-infection after treatment in schoolchildren from rural KwaZulu-Natal/South-Africa

**DOI:** 10.1186/1471-2334-4-27

**Published:** 2004-08-13

**Authors:** Elmar Saathoff, Annette Olsen, Jane D Kvalsvig, Chris C Appleton

**Affiliations:** 1 Department of Nutrition, Harvard School of Public Health, 665 Huntington Avenue, Boston, MA 02115 USA; 2 Danish Bilharziasis Laboratory, Jaegersborg Allé 1D, DK-2920 Charlottenlund, Denmark; 3 Child, Youth and Family Development, Human Sciences Research Council, Private Bag X07, Dalbridge, 4014, South Africa; 4 School of Life and Environmental Sciences, University of KwaZulu-Natal, Durban 4041, South Africa

## Abstract

**Background:**

Geohelminth infection is a major health problem of children from rural areas of developing countries. In an attempt to reduce this burden, the Department of Health of the province of KwaZulu-Natal (KZN) established in 1998 a programme for helminth control that aimed at regularly treating primary school children for schistosomiasis and intestinal helminths. This article describes the baseline situation and the effect of treatment on geohelminth infection in a rural part of the province.

**Methods:**

Grade 3 schoolchildren from Maputaland in northern KZN were examined for infections with hookworm, *Ascaris lumbricoides*, and *Trichuris trichiura*, treated twice with 400 mg albendazole and re-examined several times over one year after the first treatment in order to assess the impact of treatment and patterns of infection and re-infection.

**Results:**

The hookworm prevalence in the study population (83.2%) was considerably higher than in other parts of the province whereas *T. trichiura *and especially *A. lumbricoides *prevalences (57.2 and 19.4%, respectively) were much lower than elsewhere on the KZN coastal plain. Single dose treatment with albendazole was very effective against hookworm and *A. lumbricoides *with cure rates (CR) of 78.8 and 96.4% and egg reduction rates (ERR) of 93.2 and 97.7%, respectively. It was exceptionally ineffective against *T. trichiura *(CR = 12.7%, ERR = 24.8%). Re-infection with hookworm and *A. lumbricoides *over 29 weeks after treatment was considerable but still well below pre-treatment levels.

**Conclusion:**

High geohelminth prevalences and re-infection rates in the study population confirm the need for regular treatment of primary school children in the area. The low effectiveness of single course albendazole treatment against *T. trichiura *infection however demands consideration of alternative treatment approaches.

## Background

Geohelminth infections are among the most prevalent diseases in developing countries. Children are the group with the highest prevalences and infection intensities and are also very vulnerable to the effects of worm infection. These include nutritional deficiencies [1,2] and impaired physical and mental development [3-5], resulting in additional insults to an already disadvantaged group. Thus, even though the proportion of cases that develops clinical disease is relatively low, the enormous number of infections warrants attention to this public health problem [6].

Consequently in 1998 the KwaZulu-Natal (KZN) Department of Health initiated a pilot helminth control programme which aimed to regularly apply treatment for intestinal helminths and schistosomiasis to primary school children [7]. This group was treated without prior screening of infection status. The rationale behind this and similar programmes elsewhere is not to eliminate infection, since this could most likely only be achieved by a combination of health education, improvements in sanitation, population chemotherapy and general development which would presently overburden most developing countries. Instead the aim is to keep infection intensities in this vulnerable age group low in order to prevent serious morbidity [8,9].

Our objectives were to describe the initial pattern of geohelminth infection, assess the impact of treatment with 400 mg albendazole and to monitor re-infection after treatment in order to develop recommendations for further local and regional control efforts.

## Methods

### Study area and population, treatment and ethics

**Figure 1:**
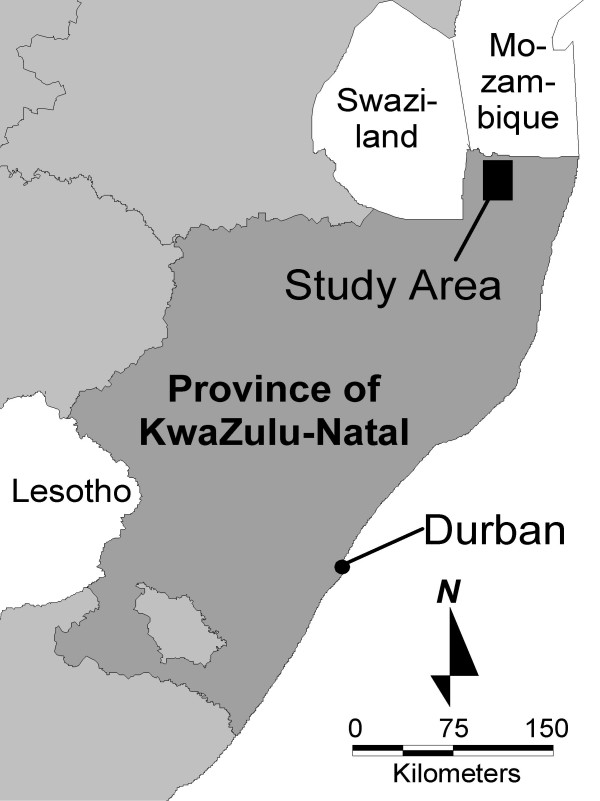
Location of the study area in northern KwaZulu-Natal

**Figure 2:**
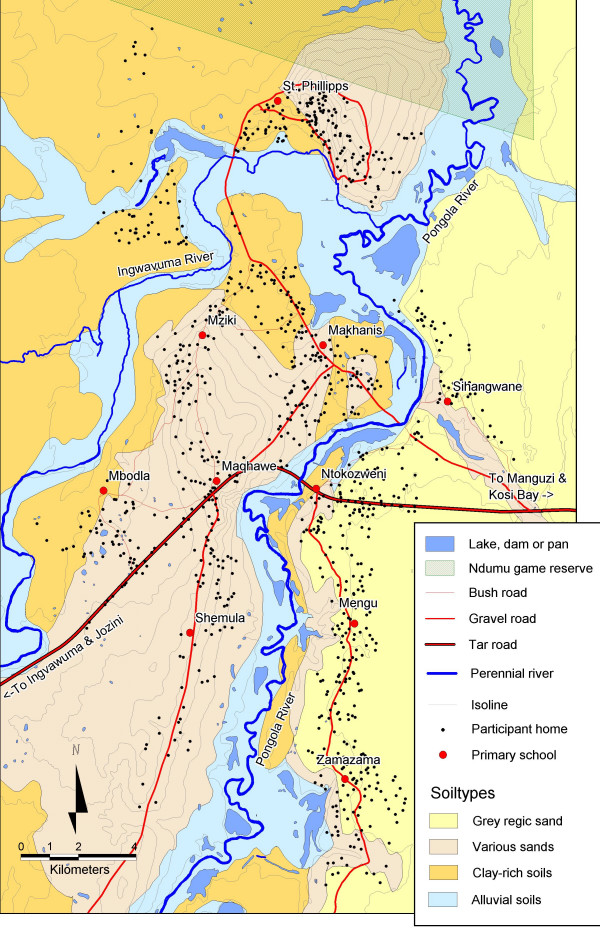
Map of the study area

**Figure 3:**
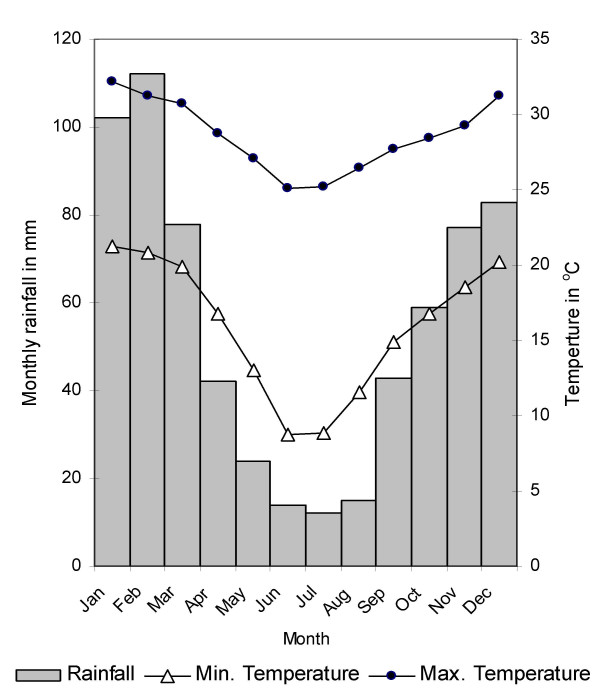
**Long-term monthly averages of rainfall and temperature in the area. **Data from 1966 to 1990 for Makatini research station, about 30 km south of the study area [35].

The study was conducted in central Ingwavuma district in northern KwaZulu-Natal (Figure 1). This area was selected because of its high geohelminth prevalences [10]. It is situated on both sides of the perennial Pongola river (Figure 2), covering approximately 28 × 16 km. The climate in the area is tropical to subtropical with a hot and wet summer (November – February) and a cooler and dry winter (June – August) (Figure 3).

The study population was limited to one grade in order to keep disturbance of the school routine low. Grade 3 was selected because it should represent the infection situation in a primary school relatively well [11]. All pupils attending grade 3 at the start of the study in all ten primary schools in the area were eligible for participation with one exception: during the baseline survey two out of five and one out of four grade 3 classes in two large schools had to be excluded due to time constraints. These classes were however included during treatments and successive surveys. Otherwise only children who refused to participate or who were absent or unable to produce a specimen during each of our repeated visits were not included in the analysis of the respective surveys due to lack of data. They were however included for those parts of the study where they participated in order not to increase bias due to the likely difference in infection status between absentees and pupils who attended school [12]. High rates of absenteeism during treatment and stool collections thus result in variable sample sizes which are therefore reported together with the results. Characteristics of the pupils who took part in the first survey are given in Table 1.

Treatment in all primary schools in the entire district was carried out by school nursing teams from the two local hospitals as part of the helminth control programme. Consenting children from all grades were treated for intestinal helminths with 400 mg albendazole (Zentel^®^, SmithKline Beecham). Treatment in the studied schools was administered in April and in October 1998. The study team assisted the nurses with treatment and also recorded those of the study population who were treated and those who were not. During the first round of treatment the pupils were also treated for schistosomiasis with 40 mg/kg praziquantel (Biltricide^®^, Bayer). However, results regarding schistosomiasis will be reported elsewhere. After the end of the study the participants were included in the normal treatment routine of the control programme.

Ethical clearance was obtained from the Ethics Committee of the Faculty of Medicine of the University of Natal/Durban and the study was also approved by the Central Medical Ethics Committee in Denmark. Before the onset of the study, information meetings were held with the staff and parents of the schools in the study. At these meetings informed consent was obtained from the parents and the staff were asked for their unpaid co-operation. The children were asked for their consent directly before the first specimen collection.

### Specimen collection and processing

After an initial survey to monitor the infection situation at baseline, two separate treatments were each followed by a survey to monitor treatment success 3 weeks later. Re-infection was assessed 16 weeks after the first and 18 and 29 weeks after the second treatment. The timing of treatments and assessments was done so as to accommodate the necessities of the treatment programme and was also restricted by the school terms.

On our visits to the schools pupils were provided with sampling equipment and asked to provide a stool specimen. Each school was visited three times during each survey in order to include children who were absent or unable to deliver a specimen on the first occasion. Apart from the pre-treatment baseline survey, where only one specimen was collected due to the above mentioned time constraints, an effort was made to obtain two stool specimens per pupil. Pupils who provided only one stool specimen (between 7 and 14% in each post-treatment survey) were nevertheless included in the analysis in order not to increase bias.

Specimens were kept cool until preparation of the slides. Duplicate 50 mg Kato-Katz cellophane thick smears were prepared from each faecal sample. They were examined by trained microscopists twice: first for hookworm eggs within one hour of preparation and later for *A. lumbricoides *and *T. trichiura *eggs within one day after preparation [13]. Accurate egg counts for both thick smears were recorded. Repeat counts by different microscopists were done on a sub sample of about 5% of the slides for quality control purposes. These counts revealed no bigger discrepancies.

Diarrhoeal specimens and slides that were too dark were not examined. Instead the respective pupils were asked to provide another specimen. Infection intensities are expressed as eggs per gram of faeces (EPG) calculated as the arithmetic mean number of eggs per thick smear multiplied by 20.

### Statistics

Data were double entered into Microsoft Excel 97 and corrected for data entry errors. Data analysis was carried out in SPSS 10.0.5 for Windows.

Prevalence ratios (PR) and their 95% confidence intervals were calculated using the SPSS "Tables" procedure [14]. Cure rates (CR) and egg reduction rates (ERR) were calculated for the examinations three weeks after treatment using the formulae below [11]:


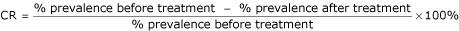



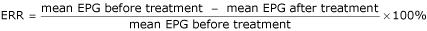


Only data from the first of the two samples we received per pupil in each post-treatment survey were used for the above calculations in order to make them comparable to the baseline survey. Otherwise differences in sensitivity between surveys would have caused misleading results. Re-infection was however calculated for both obtained samples because the main purpose was not to compare with the baseline situation but to estimate trends in re-infection reliably.

## Results

### Infection patterns at baseline

**Figure 4:**
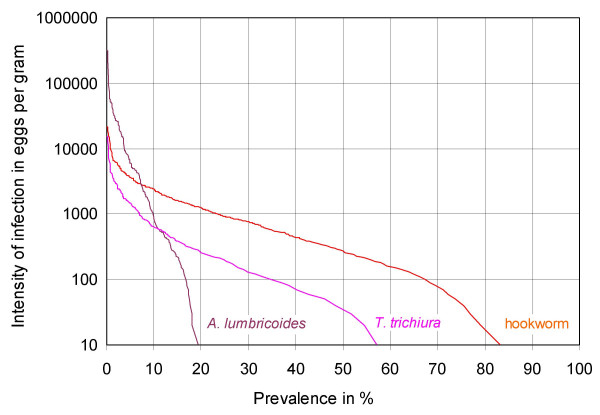
**Cumulative prevalence of geohelminth infection at baseline (n = 1017). **The prevalence of infection > = any intensity threshold of interest can be read from the percentage scale (x-axis). 10 EPG correspond to one egg on one of two slides, the minimum for a positive reading. Thus the intersection of each graph with the x-axis corresponds to the total prevalence of infection of the respective helminth.

The cumulative prevalence of all three geohelminths combined was as follows: At baseline 90.0% of the pupils were infected with one or more species, 55.9% were infected with two or more species and 30.9% of the children were infected with all three geohelminths. Figure 4 shows the intensity distribution of the three helminths in the study population and Table 1 demonstrates significant differences between the sexes for *A. lumbricoides *infection. With a PR of 0.71, males were about 30% less likely to be *Ascaris *infected than females and they also had lower infection intensities. The prevalence and intensity of hookworm infection were slightly (but nevertheless statistically significantly) higher in male pupils and the prevalence of *T. trichiura *was nearly identical in the two groups.

### Treatment

With more than 96% of the infected children cured three weeks after the first treatment and the total egg output reduced by more than 97%, albendazole was highly effective against *A. lumbricoides *infection (Table 2). It was slightly less effective against hookworm infection, but nevertheless more than 75% of the pupils were cured after the first, and more than 90% three weeks after the second treatment, and the ERR already exceeded 90% after one treatment.

Albendazole treatment was considerably less effective for *T. trichiura *infection: even after two treatments only one third of the participants were cured, and the ERR was still below 50%.

### Re-infection

The prevalences of hookworm and *A. lumbricoides *infection at 29 weeks after the second treatment were about 40% of those measured at baseline. Mean infection intensities were still considerably below this level (Table 3). Hookworm re-infection was low during the 16 weeks after the first treatment with only 10% of those pupils who were found uninfected after treatment acquiring new infections. It was markedly higher 18 weeks after the second treatment, with 21% acquiring new infections. In contrast, *A. lumbricoides *re-infection rates during both periods were fairly similar to each other.

Due to the low treatment efficacy it is difficult to make detailed conclusions about re-infection with *T. trichiura*. It is however remarkable that prevalence and intensity of this helminth apparently decreased during the re-infection period following the first treatment and that the prevalence during the 29 weeks after the second treatment appears to have been nearly stable.

## Discussion

### Infection patterns at baseline

The hookworm prevalence in the study population was higher than in comparable populations from other parts of KZN [15,16]. The predominant type of hookworm in the area seems to be *Necator americanus *although *Ancylostoma duodenale *has also been found close to the study location[16]. Prevalences of *T. trichiura *and *A. lumbricoides *were lower than in other areas on the KZN coastal plain where they usually exceed 70% [16]. A possible explanation for the latter difference is the greater distance of our study area from the coast and the resulting decrease in rainfall [17] when compared to the narrower southern parts of the coastal plain where all schools assessed [16] were relatively close to the sea.

The patterns reported here are in agreement with those reported by SCHUTTE *et al*. [10] who surveyed 45 schools in Maputaland about twenty years earlier. They also found considerably lower *A. lumbricoides *and *T. trichiura *prevalences in four schools that were situated in the area of our study than in schools closer to the coast.

The high hookworm prevalence is probably attributable to high temperature and humidity and the sandy soils in the study area which all seem to favour hookworm transmission [18,19]. Higher *A. lumbricoides *prevalences and intensities in females when compared to males are a common occurrence in many parts of the world [20] and are often attributed to different patterns of soil contact. In this study population the higher incidence of soil eating among girls might be one reason for this difference as shown in an earlier paper [21]. However, that same article found that even when geophagy preference was adjusted for, girls were still at a higher risk for *A. lumbricoides *infection at baseline. Interestingly this was not true for re-infection after treatment where boys were more often infected than girls.

### Treatment

A review that summarises numerous studies reports a median CR of 95% for *A. lumbricoides *infection and a median ERR of over 99% after a single dose of 400 mg albendazole. The corresponding numbers for hookworm infection are just above 80% and just below 90% [22]. Our results are well in agreement with these rates for both helminths.

In contrast, the CR and ERR for *T. trichiura *infection found in our study were among the lowest documented in the literature. The above cited review reports a median CR of 38% and a median ERR of 80%. Our respective rates are only about 1/3 of these. The lowest CR found in any of the 28 studies that were analysed in the review was 4.9%, the lowest ERR (23 studies) was about 27% which is still about 2% higher then the ERR that we found in this study.

A study that was conducted in Durban only about 350 km south of the study area found that albendazole reduced the median EPG of children infected before treatment by 44.1% [23]. The same outcome measure when calculated for our data is only 31.0%. But a study that was conducted in three schools about 50 km east of the area of our study also found that single dose albendazole treatment had little effect on *T. trichiura*: 6 months after treatment the treated pupils had the same prevalence (61%) as a placebo treated control group [24].

Although the CR and ERR as measured in most field studies including ours are notoriously unreliable and can only approximately indicate the true treatment success [25], the very low success rates that we found for the treatment of *T. trichiura *infection warrant attention. It is unlikely that the low CR and ERR in our study are due to low compliance: Tablet intake was monitored by the school nurses and the study team and only pupils recorded as treated were included in the analysis. Moreover, if a considerable number of children had only pretended to take the tablets, this would also have affected the impact of treatment on hookworm and *A. lumbricoides *infection. It is also unlikely that the low efficacy is due to drug resistance because of prior treatment. There were no previous control programmes in the area, to our knowledge hospitals and clinics had not been using albendazole or any other benzimidazole until recently and benzimidazoles were also not available in local shops.

High infection intensities, as they are frequently found in schoolchildren, are often mentioned in the literature as a reason for low cure rates [22,26] and this may partly explain our results. As expected the CR (but not the ERR) in our population was a lot lower in children with high pre-treatment intensities of infection than in children with lower intensities (data not shown), and in a school-based study on Pemba Island, where intensity and prevalence rank among the highest in the world, the CR for *T. trichiura *was only 10.5% [27]. But even in this population the geometric mean ERR including uninfected children was 73.3% which is in good agreement with the above cited median ERR of 80% [22] but about twice as high as the same measure for our study (36.1%). Furthermore, although it is obvious that high infection intensities can result in lower CRs, it is not clear why they should also negatively influence the ERR.

Currently there are no drugs available that are highly effective against *T. trichiura *infection as single dose treatments, but other studies show that two or three repeated doses of albendazole on consecutive days are more effective than a single dose [24,26,28,29]. It may be worthwhile to test whether this is also the case in the study area. If applied intermittently with single dose treatments this would increase drug costs but not necessarily increase the workload for the school nursing teams, as the additional doses could be administered by teachers, which is common practice in other control programmes [6,30].

The proportion of children treated in our study can not be regarded as representative for the control programme in general, because study participants were more informed about it than their schoolmates. Therefore it was not analysed statistically. However, it should be noted that although only seven pupils openly refused to be treated, absenteeism was unusually high during the first round of treatment. This improved greatly in the second treatment, when – according to the opinion of school staff – pupils and their parents had realised that treatment was beneficial.

### Re-infection

In accordance with the pre-treatment infection levels, hookworm re-infection over 29 weeks after the second treatment was relatively high and thus confirms the need for regular treatment. The moderate re-infection with *A. lumbricoides *is also in accordance with the pre-treatment situation.

The markedly higher re-infection with hookworm during the 18 weeks after the second treatment when compared to the 16 weeks after the first treatment is most likely only partly due to the slightly longer re-infection period. The difference in season could also be important [19,31]: The 16 weeks' period (April to August) fell into the relatively cool and very dry winter, whereas the 18 weeks after the second treatment (October to February) fell into the hot and humid summer. Contrastingly the absence of a clear difference between these two periods regarding re-infection with *A. lumbricoides *is not too surprising. As opposed to hookworm that is restricted to the humid tropics and subtropics, this helminth also occurs in temperate climates [20]. Its eggs are relatively robust and are thus less dependent on suitable weather conditions than the fragile free-living hookworm larvae.

Although the low efficacy of treatment for *T. trichiura *complicates conclusions, the data seem to indicate that re-infection was low. Therefore, if a more effective treatment approach could be utilised, the resulting decrease in prevalence and intensity of *T. trichiura *infections might be relatively long-lasting.

## Conclusion

This study has shown that the high prevalence and intensity of geohelminth infection necessitate regular treatment of primary schoolchildren in the study area according to WHO criteria [6]. This is underlined by the fact that our baseline data and those from Schutte et al. are little different despite the passage of 20 years which indicates that transmission and epidemiology appear not to have changed much. This means that without intervention, geohelminths will likely remain a problem in the area.

Because high prevalences in primary schoolchildren may also indicate relatively high community prevalences [32] it should be investigated whether other high risk groups e.g. pre-schoolers and pregnant women also need treatment for geohelminths [33,34].

Our study has also demonstrated that a single course of treatment with 400 mg of albendazole is a powerful tool to control hookworm and *A. lumbricoides *infection, but that it is much less effective for the treatment of *T. trichiura *infection in the study area. It would be important to find out whether albendazole treatment is similarly ineffective against *T. trichiura *in other parts of the province and to test the effectiveness of treatment alternatives.

To answer the question whether hookworm transmission is really seasonal as this study seems to suggest would necessitate a cohort study over at least one year. Nevertheless it might be worthwhile to investigate when in the year transmission is highest in order to schedule treatment accordingly.

## Competing interests

None declared.

## Authors' contributions

ES conceived of the study and designed it with input from all authors. He conducted the field work with contributions from the other authors, did the statistical analysis and drafted the manuscript. All authors contributed to the final version of the manuscript and read and approved it.

## Pre-publication history

The pre-publication history for this paper can be accessed here:



## Figures and Tables

**Table 1:** Prevalence and intensity (EPG) of geohelminth infection at baseline

	Males	Females	M/F Ratio* (95%CI or *P*)
n =	445	572	
Median Age (Inter quartile range)	11.2 (10.1–12.3)	10.7 (9.7–11.8)	
Prevalence (%)			
Hookworm	86.5	80.6	1.07 (1.02 to 1.13)
*A. lumbricoides*	15.7	22.2	0.71 (0.54 to 0.92)
*T. trichiura*	57.3	57.2	1.00 (0.90 to 1.12)
Mean Intensity (EPG)^†^			
Hookworm	984	792	1.24 (*P *< 0.001)
*A. lumbricoides*	876	2948	0.30 (*P *= 0.008)
*T. trichiura*	240	332	0.72 (*P *= 0.671)

* Male to Female ratios for prevalence and mean intensity of infection and 95% confidence interval (for prevalence) or *P*-value of 2-sided Mann-Whitney-U-test (for differences in mean EPG). ^† ^Arithmetic mean EPG including uninfected pupils.

**Table 2:** Cure rates and egg reduction rates at three weeks after the first and after the second treatment with 400 mg albendazole (n = 592)*

	Hookworm	*A. lumbricoides*	*T. trichiura*
Prevalence (%)			
Baseline Survey	82.9	22.0	59.8
After Treatment 1	17.6	0.8	52.2
After Treatment 2	5.7	0.5	39.9
Cure rate (%)			
1 Treatment	78.8	96.4	12.7
2 Treatments	93.2	97.7	33.3
Arithmetic Mean EPG^†^			
Baseline Survey	881	2213	319
After Treatment 1	60	52	240
After Treatment 2	11	3	166
ERR (%)			
1 Treatment	93.2	97.7	24.8
2 Treatments	98.8	99.8	47.8

* Including only pupils who participated in both treatments, the baseline survey and in the surveys directly after the first and second treatment and calculated only using the first of two samples that were obtained in the post-treatment surveys. ^† ^Calculated including uninfected children.

**Table 3:** Development of prevalence and intensity after one and after two rounds of treatment with 400 mg albendazole*

	Post-Treatment 1^‡^	Re-infection 1	Post-Treatment 2^‡^	Re-infection 2a	Re-infection 2b
n =	801	790	803	739	731
Time of survey	May/Jun 98	Aug 98	Oct/Nov 98	Feb 99	Apr/May 99
Weeks since last treatment	3	16	3	18	29
Prevalence (%)					
Hookworm	21.2	24.8	10.1	24.6	36.7
*A. lumbricoides*	1.0	4.3	0.7	3.0	8.6
*T. trichiura*	54.6	50.9	44.0	44.8	43.4
Mean intensity (EPG)^†^					
Hookworm	77	184	14	65	139
*A. lumbricoides*	55	115	3	59	351
*T. trichiura*	227	215	116	174	175

* Included are all pupils who participated in both treatments (n = 864) and in the respective survey (n given in table). Calculated using both samples that were obtained in each survey. ^† ^Arithmetic mean EPG for all pupils including uninfected. ^‡ ^The two treatments were administered in April and October 1998 respectively.
